# Placebo-arm response rates for pain outcomes in chronic pancreatitis: a systematic review and meta-analysis of randomized controlled trials

**DOI:** 10.3389/fphys.2026.1954055

**Published:** 2026-10-22

**Authors:** Tareq Alsaleh, Prachi Mann, Aleena Majeed, Syed Hasaan Rahman, John George

**Affiliations:** 1 Internal Medicine, AdventHealth Orlando, Orlando, FL, United States; 2 Kansas City University College of Medicine, Kansas City, MO, United States; 3 Gastroenterology and Hepatology, AdventHealth Orlando, Orlando, FL, United States; 4 Pancreas Center, AdventHealth Orlando, Orlando, FL, United States

**Keywords:** chronic pancreatitis, meta - analysis, pain, placebo, randomized controlled trials

## Abstract

**Background:**

Improvement in the placebo arm can influence chronic pancreatitis (CP) pain trials, yet contemporary estimates of placebo-arm response are limited. We aimed to quantify placebo-arm pain response rates in medical placebo-controlled randomized controlled trials (RCTs) of CP-related abdominal pain.

**Methods:**

PubMed, Web of Science, Embase, and Scopus were searched from inception to October 2025 for placebo-controlled RCTs reporting pain response among placebo-treated patients with CP. Crossover trials, procedural or device-based sham-controlled trials, and studies without extractable placebo-arm pain-response data were excluded. The primary outcome was the pooled proportion of placebo-treated patients achieving pain response according to study-specific definitions. Random-effects meta-analysis of single proportions was performed. Subgroup analyses categorized endpoints as partial pain improvement or complete pain resolution. Risk of bias was assessed using Cochrane RoB 2.

**Results:**

Seven RCTs including 321 placebo-treated patients, 320 of whom contributed pain-response data, met inclusion criteria. Follow-up ranged from 3 to 26 weeks. One study was judged low risk of bias and six had some concerns; no study was high risk. The pooled placebo-arm pain response rate was 26% (95% CI, 17%–36%), with heterogeneity (I² = 74.8%). Response rates varied with endpoint stringency, although the difference between subgroups was not statistically significant (P = 0.18): 31% (95% CI, 18%–46%; I² = 73.7%) in trials using partial pain improvement criteria and 20% (95% CI, 12%–29%; I² = 42.1%) in trials requiring complete pain resolution. Leave-one-out sensitivity analysis did not materially alter the estimate.

**Conclusions:**

One-quarter of placebo-treated patients in CP RCTs experience pain response. Endpoint stringency may influence observed placebo-arm response and should be considered in sample-size estimation, outcome selection, and interpretation of CP pain trials.

## 1 Introduction

Chronic pancreatitis (CP) is a progressive fibro-inflammatory disorder characterized by irreversible pancreatic damage and exocrine and/or endocrine insufficiency. Abdominal pain is the predominant manifestation of CP, affecting approximately 80–90% of patients and contributing substantially to healthcare utilization, disability, and impaired quality of life (Drewes et al., 2017). CP-related pain is multifactorial and involves mechanical, neuroinflammatory, and central pain-processing abnormalities, including central sensitization and impaired descending pain modulation (Drewes et al., 2008). This complexity may explain the poor correlation between pancreatic morphology and pain severity and the heterogeneity in treatment response.

Given the multifactorial nature of CP-related pain and the variability in treatment response, the evaluation of new treatments for pain in CP relies on well-designed placebo-controlled randomized trials. These trials employ pharmacologic agents tested against an inert medical placebo and procedural or device-based interventions, such as endoscopic therapy and device-based neuromodulation, tested against a sham control. These control arms have different non-specific effects, so they are not interchangeable baselines. Improvement in the placebo arm is a well-recognized phenomenon in chronic pain trials and can substantially influence trial outcomes. A fundamental component of robust trial design is accurate estimation of the placebo-arm response rate, which serves as the baseline against which active treatment effects are measured. Underestimation of this response rate may lead to underpowered studies and false-negative results (Schulz and Grimes, 2005). Knowledge of the expected placebo-arm response rate is therefore critical for accurate sample size calculation and interpretation of trial results.

A 2012 meta-analysis by Capurso et al. remains the only formal evaluation of placebo-arm response in CP pain trials and reported a pooled placebo remission rate of approximately 20%, with substantial heterogeneity related to multicenter design, short run-in periods, and crossover trials without washout periods (Capurso et al., 2012). Since that analysis, several additional placebo-controlled randomized trials evaluating therapies such as antioxidants, pancreatic enzyme preparations, and neuromodulators have expanded the available evidence base.

Given the expanded evidence base and lack of standardized pain management in CP, an updated meta-analysis of placebo-arm response is both timely and necessary. We conducted a systematic review and meta-analysis of medical placebo-controlled randomized trials to quantify the pooled placebo-arm pain response in CP-related abdominal pain using contemporary trial data. We additionally examined placebo-arm response according to outcome stringency, distinguishing studies evaluating partial pain improvement from those requiring complete pain resolution. By characterizing the magnitude of placebo-arm response, this study may inform interpretation of prior trials, improve estimation of treatment effects, support clinical decision-making, and guide the design of future randomized studies of therapies for CP-related abdominal pain, including the choice between a medical placebo and a sham control.

## 2 Methods

### 2.1 Protocol and registration

This meta-analysis follows the updated preferred reporting items for systematic reviews and meta-analyses (PRISMA) 2020 statement. The review protocol was not registered. The eligibility criteria, the primary outcome, and the subgroup analysis by endpoint stringency were prespecified before data extraction.

### 2.2 Search strategy and study selection

A systematic search of PubMed, Web of Science, Embase, and Scopus was performed from inception to October 2025 for placebo-controlled randomized controlled trials (RCTs) reporting pain response in patients with chronic pancreatitis (CP).

The inclusion criteria comprised randomized, placebo-controlled trials, as full-text publications or conference proceedings, that reported the incidence of pain response in CP patients receiving medical placebo. Exclusion criteria were as follows: crossover RCTs, RCTs using procedural, device-based, or surgical sham controls, RCTs not reporting pain response in the placebo arm, overlapping cohorts without extractable independent data, and non-randomized studies. Relevant non-crossover sham-controlled procedural or neuromodulation trials identified during screening or citation review were discussed narratively when they provided contextual information relevant to placebo and sham responses in CP pain trials.

Procedural and device-based sham-controlled trials were not eligible for quantitative synthesis because medical placebo and sham controls differ substantially for several reasons. A control must match the intervention being investigated, and an inert capsule cannot control for the effects of an endoscopic, surgical, or device-based procedure. In addition, vulnerability to unblinding differs. A pill placebo can be closely matched to an active drug. Sham procedures, in contrast, may be unblinded by sedation, recovery course, or procedural sensation. Furthermore, the non-specific effects captured differ in kind. Beyond the expectation of benefit, a sham control incorporates procedural ritual, sedation or anesthesia, therapeutic contact time, and the expectations generated by an invasive intervention. Invasive sham procedures also expose participants to risk without prospect of benefit, which constrains sample size, eligibility, and follow-up duration and may require crossover to active treatment after the primary endpoint. Importantly, patients willing to accept randomization to an invasive sham may differ systematically from those enrolling in drug trials. Pooling the two control conditions would therefore produce a baseline that applies to neither design.

Overlapping cohorts were identified by cross-referencing institutions, study periods, and authorship. We prioritized peer-reviewed full-text articles over conference abstracts to ensure data quality. In cases of overlapping full-text RCTs, the article with the largest sample size or most complete placebo-arm pain response data was included as the primary data source.

We utilized a combination of medical subject heading terms and free-text keywords related to CP, abdominal pain, and placebo. No geographic restrictions were used, and recursive citation searching was used to identify other eligible studies. The full search strategy is provided in the **Supplementary Materials**. Two investigators, T.A. and P.M., independently screened titles, abstracts, and full texts, with discrepancies resolved by a third investigator, A.M.

### 2.3 Data extraction and outcome measures

Two independent investigators, T.A. and P.M., extracted data into a standardized spreadsheet, with discrepancies resolved by a third investigator, A.M. We collected data from each eligible study, including author name, publication year, country, study design, intervention type, number of patients, placebo-arm sample size, demographics, disease characteristics, follow-up duration, definition of pain response, and placebo-arm pain response events.

The primary outcome was the proportion of patients in the placebo arm who achieved pain response. This proportion is referred to throughout as the placebo-arm pain response, which describes the response observed in that arm. This is an important distinction from placebo-induced response. The study-specific definition of pain response was retained for the primary analysis. To address heterogeneity in endpoint stringency, subgroup analysis was performed by categorizing outcomes as either partial pain improvement or complete pain resolution. Partial pain improvement included improvement-based criteria such as patient global impression of change, clinician-assessed improvement, or prespecified percentage reduction in pain scores. Complete pain resolution included studies requiring pain freedom or complete remission.

### 2.4 Quality assessment

Risk of bias in the included studies was assessed independently by two reviewers, T.A. and P.M., using the Cochrane Risk of Bias 2 tool for randomized trials. A third reviewer, A.M., resolved discrepancies. Risk of bias was assessed across the following domains: randomization process, deviations from intended interventions, missing outcome data, outcome measurement, and selection of the reported result (Sterne et al., 2019).

### 2.5 Statistical analysis

The primary analysis pooled placebo-arm response proportions using a random-effects model for single proportions. Proportions were stabilized using the Freeman-Tukey double arcsine transformation, and between-study variance was estimated with the DerSimonian–Laird method. Studies were weighted by inverse variance, incorporating the between-study variance. Pooled estimates were back-transformed to the proportion scale using the inverse Freeman-Tukey transformation with the harmonic mean of the study sample sizes, and confidence intervals were derived from the normal distribution. A 95% prediction interval was calculated to describe the response expected in a future trial. Illustrative sample sizes were calculated for a two-sided comparison of two independent proportions, with an α of 0.05, 80% power, 1:1 allocation, the normal approximation without continuity correction, and no allowance for attrition. Subgroup analyses used the same model, with between-study variance estimated separately within each subgroup. Subgroups were compared using a test for subgroup differences based on the random-effects estimates. Pooled estimates were expressed as proportions with 95% confidence intervals (CIs). Statistical heterogeneity was assessed using Cochran’s Q test and quantified with the I² statistic, interpreted in accordance with the Cochrane Handbook (Higgins et al., 2024).

Continuous variables reported as medians and ranges/interquartile ranges (IQRs) were converted to means using established methods (McGrath et al., 2020). Mean values from individual studies were aggregated as weighted averages, with the number of patients in each study used as weights. Sensitivity analyses were performed by assessing the impact of individual study exclusion on the pooled estimate. Additional sensitivity analyses were performed using the restricted maximum likelihood estimator in place of the DerSimonian–Laird estimator (DerSimonian and Laird, 1986) and using a logit transformation fitted as a random-intercept logistic model. Publication bias was not formally assessed because fewer than 10 studies were included. Statistical analyses were performed using R version 4.5.2 and the meta package.

## 3 Results

### 3.1 Search results, population, and study characteristics

The literature search identified 1,026 records, from which 416 duplicates were removed, leaving 610 unique titles and abstracts for screening. Of these, 69 full-text articles were assessed for eligibility, and ultimately, seven studies met the inclusion criteria for quantitative synthesis (**Figure 1**) (Shiratori et al., 2002; Bhardwaj et al., 2009; Olesen et al., 2011; Talukdar et al., 2016; Sureshkumar et al., 2021; Hart et al., 2024; Rana et al., 2026).

**Figure 1:**
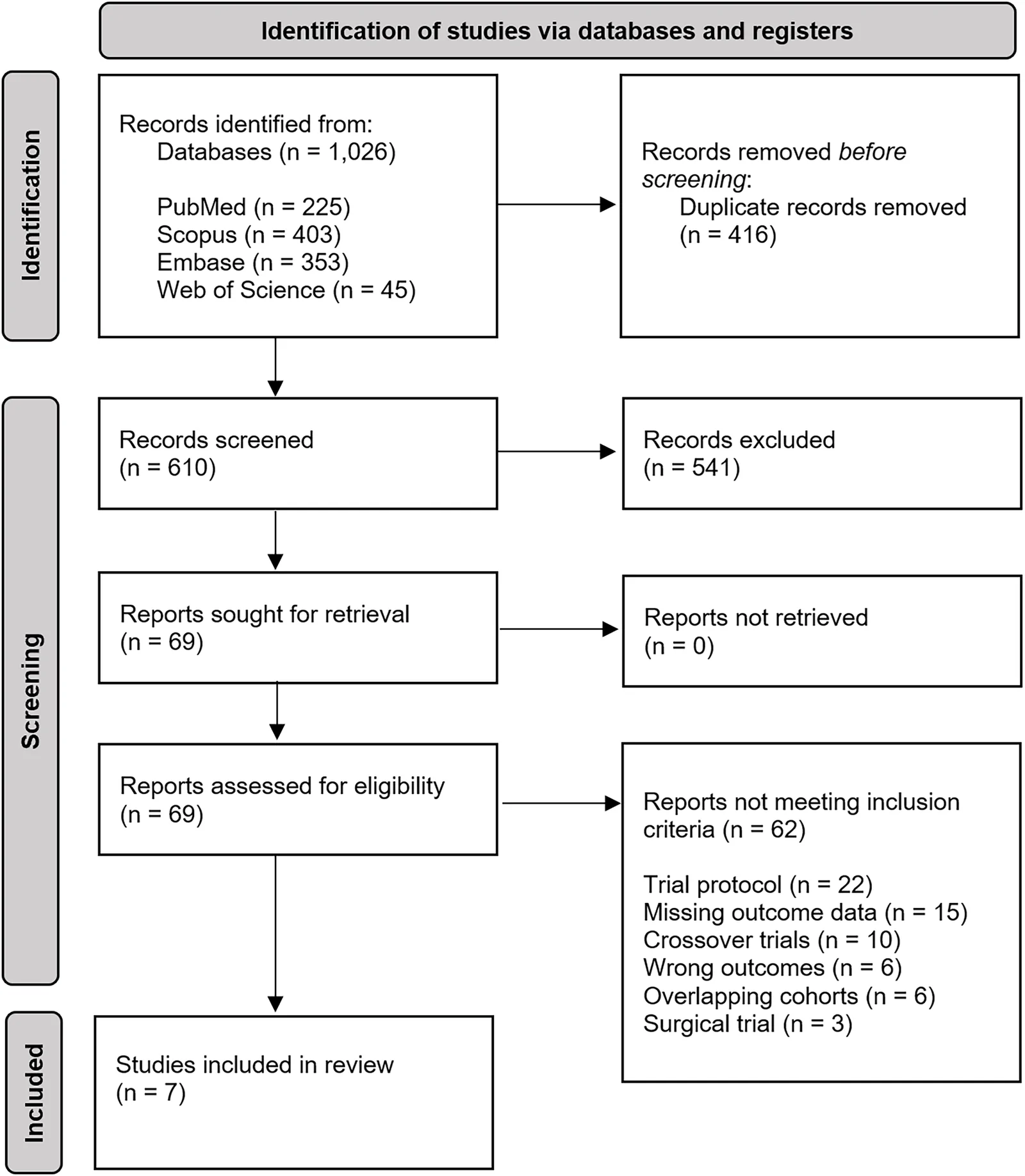
PRISMA flowchart summarizing the literature search process with the final inclusion of seven eligible randomized-controlled trials.

The seven studies included 321 placebo-treated patients with CP-related pain. Pain-response data were available for 320 of these patients, because one patient in the placebo arm of Rana 2025 was lost to follow-up. The mean age was 43.7 years, and 67% were male. Patients with diabetes comprised 24% of the population, while 29.8% were active smokers in the four trials reporting smoking status. Most cases of CP were idiopathic (63.6%), and the mean disease duration was 58.2 months. Follow-up duration ranged from 3 to 26 weeks across the trials (**Table 1**).

**Table 1:** Baseline study characteristics.

Study	Location	Multi/Single center	Patient population	Sample size, n	Age, mean (SD)	Males, n (%)	Diabetes, n (%)	Idiopathic	Alcohol	Active smoker, n (%)	Active alcohol, n (%)	Steatorrhea, n (%)	On PERT, n (%)	Antioxidant therapy, n (%)	Follow-up duration, weeks
Bhardwaj 2009	India	Single-center	Patients with chronic pancreatitis	56	29.6 (9.3)	39 (70)	8 (14)	41	15	14 (25)	NR	12 (21)	56 (100)	NR	26
Hart 2024	USA	Multi-center	Patients with chronic pancreatitis	67	51 (11.7)	38 (56.7)	16 (23.9)	48	19	15 (22.4)	NR	NR	46 (68.7)	NR	4
Olesen 2011	Denmark and The Netherlands	Multi-center	Patients with chronic pancreatitis	30	55 (12)	19 (63)	10 (33)	11 (37)	17 (57)	22 (73)	11 (37)	NR	13 (43)	NR	3
Rana 2025	India	Single-center	Patients with chronic pancreatitis	25	28.8 (10.70)	17 (68)	9 (36)	23	2	2 (8)	0 (0)	10 (40)	19 (76)	10 (40)	12
Shiratori 2002	Japan	Multi-center	Patients with chronic pancreatitis	53	59 (14)	34 (64)	9 (17)	31	18	NR	NR	NR	28 (52.8)	NR	4
Sureshkumar 2021	India	Single-center	Patients with chronic pancreatitis	45	45.2	36 (80)	19 (42.2)	16	29	NR	NR	NR	NR	NR	26
Talukdar 2016	India	Single-center	Patients with chronic pancreatitis	45	31.6 (3.6)	32 (71.1)	6 (13.3)	34	11	NR	NR	10 (22.2)	NR	NR	8

SD, Standard deviation; PERT, Pancreatic enzyme replacement therapy; NR, Not reported. In Rana 2025, 25 patients were randomized to placebo and 24 had pain-response data available.

The RCTs were conducted in India (k = 4) (Bhardwaj et al., 2009; Talukdar et al., 2016; Sureshkumar et al., 2021; Rana et al., 2026), the USA (k = 1) (Hart et al., 2024), Japan (k = 1) (Shiratori et al., 2002), and Denmark/the Netherlands (k = 1) (Olesen et al., 2011). Three RCTs were multicenter (Shiratori et al., 2002; Olesen et al., 2011; Hart et al., 2024), while the remaining four were single-center studies (Bhardwaj et al., 2009; Talukdar et al., 2016; Sureshkumar et al., 2021; Rana et al., 2026).

In the Cochrane RoB 2 assessment of the placebo-arm pain-response outcome, one trial was at low risk of bias, six had some concerns, and no trial was at high risk. The most common reasons for some concerns were incomplete reporting in older studies, limited handling of post-randomization exclusions, and possible functional unblinding in pregabalin-containing trials due to recognizable central nervous system adverse effects (**Table 2**).

**Table 2:** Risk of bias assessment per Cochrane’s Risk of Bias 2 tool for randomized-controlled trials.

Study	D1: randomization	D2: deviations	D3: missing data	D4: outcome measurement	D5: selective reporting	Overall	Short rationale
Bhardwaj 2009	Low	Low	Some concerns	Low	Low	Some concerns	Good randomization/blinding. Concern mainly from attrition before final analysis.
Hart 2024	Low	Low	Low	Low	Low	Low	Modern multicenter blinded RCT with ITT analysis, low attrition, and prespecified outcomes.
Rana 2025	Low	Low	Low	Some concerns	Low	Some concerns	Good allocation and low missingness. Pregabalin side effects may have weakened blinding for subjective pain outcomes.
Sureshkumar 2021	Low	Low	Low	Some concerns	Some concerns	Some concerns	Good randomization/blinding and complete follow-up. Concern from subjective outcomes and less clear prespecification of complete pain resolution.
Talukdar 2016	Low	Low	Low	Some concerns	Low	Some concerns	Well-randomized blinded trial. Concern mainly from subjective pain outcomes and possible functional unblinding.
Olesen 2011	Low	Low	Low	Some concerns	Low	Some concerns	Strong methods and low missingness. Pregabalin-related CNS effects could partially unblind subjective outcome assessment.
Shiratori 2002	Some concerns	Low	Some concerns	Some concerns	Some concerns	Some concerns	Older trial with limited reporting of randomization, post-randomization exclusions, and less rigorous pain-response measurement.

RCT, Randomized controlled trial; ITT, Intention-to-treat; CNS, Central nervous system.

### 3.2 Meta-analysis results

#### 3.2.1 Primary outcomes: pain response

Across the seven trials, “pain response” was variably defined and included sustained pain freedom for at least 6 months, patient-reported global improvement on PGIC, and clinician-assessed pain improvement after treatment (**Table 3**).

**Table 3:** Definitions of pain response in the included trials.

Study	Definition of response
Bhardwaj 2009	At least 6 months pain-free
Hart 2024	Very much or much improvement in PGIC
Olesen 2011	30% change in the worst pain intensity score (averaged over 7 days of the week) from baseline for 2 of the 4 weeks of double-blind treatment.
Rana 2025	Moderate or better improvement in PGIC
Shiratori 2002	Clinical improvement of pain after treatment
Sureshkumar 2021	Complete pain resolution
Talukdar 2016	Complete pain resolution

PGIC, Patient Global Impression of Change.

Using these trial-specific definitions, the pooled placebo-arm pain response rate was 26% (95% CI, 17–36), with substantial heterogeneity (I² = 74.8%) (**Figure 2**). The 95% prediction interval was 2% to 61%.

**Figure 2:**
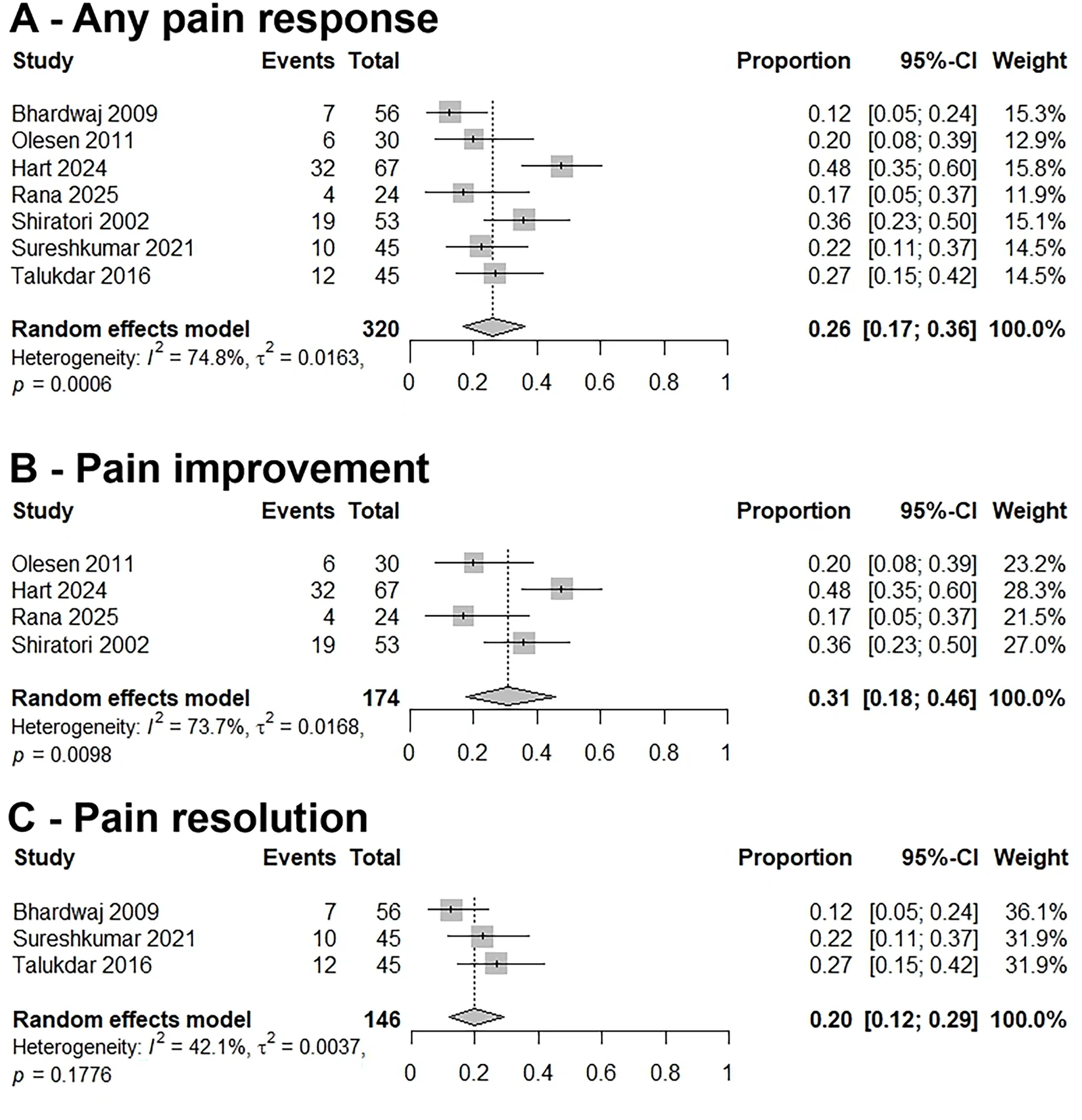
Forest plots summarizing the pooled rates of pain response **(A)**, pain improvement **(B)**, and pain resolution **(C)** in patients receiving placebo for chronic pancreatitis.

#### 3.2.2 Subgroup analysis

Subgroup analyses were conducted by response stringency. When response was defined as partial pain improvement, the pooled rate was 31% (95% CI, 18–46; I² = 73.7%). When response was defined as complete pain resolution, the pooled rate was 20% (95% CI, 12–29), with lower heterogeneity (I² = 42.1%) (**Figure 2**). Four trials contributed to the partial pain improvement subgroup and three to the complete pain resolution subgroup. The difference between subgroups was not statistically significant (Q = 1.83, df = 1, P = 0.18).

### 3.3 Validation of meta-analysis results

#### 3.3.1 Sensitivity analysis

A leave-one-out sensitivity analysis was performed to measure the impact of individual study omission on the pooled placebo-arm pain response rate. No individual study materially changed the pooled estimate (**Table 4**). The pooled estimate was also similar using the restricted maximum likelihood estimator (26%, 95% CI 17–36) and a random-intercept logistic model (25%, 95% CI 18–35).

**Table 4:** Leave-one-out sensitivity analysis evaluating the impact of individual study omission on the pooled primary endpoint (pain response).

Analysis (omitted study)	Pooled proportion	95% CI	I²
Omitting Bhardwaj 2009	0.29	0.20–0.39	65.40%
Omitting Olesen 2011	0.27	0.16–0.39	78.20%
Omitting Hart 2024	0.22	0.15–0.30	45.20%
Omitting Rana 2025	0.27	0.17–0.39	77.60%
Omitting Shiratori 2002	0.24	0.14–0.36	77.10%
Omitting Sureshkumar 2021	0.26	0.16–0.39	78.40%
Omitting Talukdar 2016	0.26	0.15–0.38	79.00%
Random-effects model (all studies)	0.26	0.17–0.36	74.80%

CI, Confidence interval.

#### 3.3.2 Heterogeneity

Heterogeneity was moderate-to-substantial across outcomes, indicating dispersion in effect estimates across studies.

#### 3.3.3 Publication bias

Publication bias was not assessed because of the limited number of studies (k = 7).

## 4 Discussion

This meta-analysis found a pooled placebo-arm pain response rate of 26% in CP-related abdominal pain, compared with 20% in the only prior meta-analysis by Capurso et al. (2012) Only two trials overlap between the two analyses, Bhardwaj 2009 and Olesen 2011 (Bhardwaj et al., 2009; Olesen et al., 2011), while the remaining five differed in setting, patient population and outcome definitions. Placebo-arm response rates also varied by endpoint definition, with higher rates in studies evaluating partial pain improvement (31%) than complete pain resolution (20%). The difference between subgroups was not statistically significant, and because response definitions differed and no standardized outcome measure exists, these subgroup estimates are descriptive planning figures. These findings align with broader placebo literature showing higher responses with less stringent endpoint definitions (Su et al., 2007).

These findings have implications for the design of future CP pain trials. Since significant heterogeneity was observed, no single assumption is appropriate for sample size calculation, and trials should plan across a reference range: 26% (95% CI, 17–36) overall, 31% (95% CI, 18–46) under partial pain improvement endpoints, and 20% (95% CI, 12–29) under complete pain resolution endpoints, with the sample sizes required across this range shown in **Table 5**. Underestimating control-arm response can result in underpowered studies and false-negative results, so these results can help guide future trials to achieve adequate statistical power. Importantly, the substantial heterogeneity across studies also highlights the need to standardize pain outcome definitions and adopt validated patient-reported outcome measures.

**Table 5:** Illustrative sample size per arm under different control-arm response assumptions.

Trial design and endpoint	Assumed control-arm response	Maximum possible absolute difference, percentage points	Patients per arm for a 10-percentage-point difference	Patients per arm for a 15-percentage-point difference
Medical placebo, complete pain resolution (present review)	20%	80	294	138
Medical placebo, all endpoints (present review)	26%	74	335	155
Medical placebo, partial pain improvement (present review)	31%	69	361	165
Invasive procedural sham, partial pain improvement (illustrative scenario)	60%	40	356	152
Invasive procedural sham, partial pain improvement (single trial, Talukdar et al. (2024))	90%	10	Requires 100% active-arm response	Not possible

The 20%, 26%, and 31% assumptions are the pooled estimates from this review. The 60% and 90% assumptions are illustrative, the latter taken from the single available invasive sham-controlled trial (Talukdar et al., 2024). Required sample size peaks near a control-arm response of 50%, so at higher control-arm response the binding constraint is the maximum possible absolute difference rather than the number of patients.

The choice between a medical placebo and a sham control has direct consequences for the expected control-arm response, and our estimates anchor the medical placebo side of that choice. Sham devices and procedures have produced larger placebo responses than inert pills in other pain conditions (Kaptchuk et al., 2006; Meissner et al., 2013). In the sham-controlled RCT by Talukdar et al. evaluating extracorporeal shock-wave lithotripsy (ESWL) plus endoscopic pancreatic therapy, 91% of sham-treated patients achieved ≥30% pain relief at 12 weeks (Talukdar et al., 2024). Because a ≥30% reduction meets our prespecified definition of partial pain improvement, the appropriate comparison is 91% against the pooled 31% for partial pain improvement rather than against the overall 26%. The control-arm response of a sham-controlled trial therefore cannot be assumed to approximate the medical placebo estimate (**Table 6**).

**Table 6:** Comparison of medical placebo-controlled and sham-controlled designs in randomized trials of CP-related pain.

Design feature	Medical placebo control	Sham control
Interventions evaluated	Pharmacologic agents (e.g., antioxidants, pregabalin, camostat)	Endoscopic, lithotripsy, surgical, and device-based neuromodulation interventions
What the control isolates	Pharmacologic effect of the drug beyond the non-specific effects of taking a study medication within a trial	Specific effect of the procedure or device beyond the non-specific effects of undergoing a procedure within a trial
Blinding vulnerability	An inert pill can be closely matched. Functional unblinding is possible when the active drug has recognizable adverse effects (e.g., central nervous system effects of pregabalin).	The procedure must be simulated, and operators generally cannot be blinded. Sedation, recovery course, or procedural sensation may reveal allocation.
Sources of non-specific effect	Expectation of benefit, regression to the mean, fluctuating symptom severity, increased clinical attention, and permitted analgesic cointerventions	The same factors, plus procedural ritual, invasiveness, sedation or anesthesia, and therapeutic contact time
Ethical and feasibility constraints	Minimal risk from the control itself. Larger samples and longer follow-up are feasible.	Procedural risk without prospect of benefit constrains sample size, eligibility, and follow-up. Crossover to active treatment may be required after the primary endpoint. Patients accepting randomization to sham may differ from drug-trial populations (Beard et al., 2020).
Expected control-arm response	Overall 26% (95% CI, 17–36), partial pain improvement 31% (95% CI, 18–46), and complete pain resolution 20% (95% CI, 12–29) in the present review	Not estimable from current evidence. After sham ESWL and endoscopy, 91% of patients achieved ≥30% pain relief at 12 weeks (Talukdar et al., 2024), whereas sham rTMS produced negligible change (Fregni et al., 2011).

CI, Confidence interval; ESWL, Extracorporeal shock-wave lithotripsy; Rtms, Repetitive transcranial magnetic stimulation.

With only two sham-controlled trials (k = 2), their results could not be pooled and no single conservative assumption can be recommended. In invasive procedural sham trials with partial pain improvement endpoints, control-arm response should be planned at rates approaching or exceeding the active arm in drug trials (60% and 90% in **Table 5**), not at the 26% to 31% estimates derived here. In non-invasive device trials the sham response may instead be negligible (Fregni et al., 2011), so the assumption should be tied to the invasiveness and intensity of the sham. Endpoint stringency then determines whether a procedural trial can succeed, because at a control-arm response of 91% (Talukdar et al., 2024) the maximum possible absolute difference is 9 percentage points, and a larger clinically meaningful effect cannot be demonstrated regardless of sample size. Procedural trials would therefore benefit from using stringent or objective primary endpoints, such as complete pain resolution, opioid cessation, or intervention-free survival, rather than ≥30% pain relief or PGIC. Prespecified sensitivity analyses across responder thresholds, sham run-in or enrichment designs, and follow-up beyond 12 weeks should also be considered.

Compared to a prior meta-analysis by Capurso et al. (2012), our analysis produced similar estimates despite several methodological differences. In our study, crossover trials were excluded to reduce carryover and washout-related bias. Our search extended to October 2025, resulting in a larger placebo-treated population (321 vs 202 patients). Despite these refinements, heterogeneity remained substantial (I² = 74.8%), comparable to that reported by Capurso et al. (I² = 76%). Much of this dispersion was attributable to a single trial, because omission of Hart 2024, in which 48% of placebo-treated patients improved on PGIC, reduced I² from 74.8% to 45.2% and lowered the pooled estimate to 22% (**Table 4**). Likely sources of this heterogeneity include differences in response definitions, follow-up duration, single-center compared with multicenter conduct, etiology, and trial design, because the two trials with the highest response were dose-finding trials with higher odds of receiving active treatment.

Pain in CP involves neuroinflammatory and central pain processing mechanisms, including central sensitization and impaired descending pain modulation (Drewes et al., 2008; Olesen et al., 2010). These mechanisms overlap with pathways involved in placebo analgesia and expectation-mediated pain modulation (Olesen et al., 2010). In addition, improvement in the placebo arm likely reflects non-specific factors, including regression to the mean, the natural history of the disease, fluctuating symptom severity, increased clinical attention during trial participation and permitted analgesic cointerventions (Hróbjartsson and Gøtzsche, 2010). These influences cannot be separated from expectation-mediated effects, because no included trial had a no-treatment arm. The estimate reported here is better described as a placebo-arm response rather than an isolated placebo effect. This does not limit its use in trial design, because sample size estimation requires the total response observed in the control arm, irrespective of mechanism.

This study provides an updated synthesis with a larger and more recent evidence base than prior analyses. Furthermore, strict inclusion criteria with exclusion of crossover studies avoids bias imposed by carryover and washout-related bias. Risk of bias was low in one trial and of some concern in six, with no trial judged at high risk. However, several limitations should be noted. Substantial residual heterogeneity persisted across analyses, and reliable meta-regression or assessment of publication bias was not possible due to the small number of studies (k = 7). In addition, individual patient-level data were unavailable, limiting assessment of predictors of placebo-arm response such as pain severity, disease duration and baseline opioid use. Finally, the predominance of studies conducted in India, where idiopathic CP is more prevalent (Garg, 2012), may limit generalizability to populations in which alcohol-related CP predominates.

Nevertheless, this study provides an updated estimate of placebo-arm pain response in CP, providing important guidance for future trial design. Larger placebo-controlled and sham-controlled trials with prespecified response assumptions are needed to evaluate emerging therapies for CP-related pain.

5.

## Data Availability

The original contributions presented in the study are included in the article/**Supplementary Material**. Further inquiries can be directed to the corresponding author.
