# Supplementary material for: Placebo-arm response rates for pain outcomes in chronic pancreatitis: a systematic review and meta-analysis of randomized controlled trials
Source: Front Physiol. 2026 Oct 22;17:1954055. doi: 10.3389/fphys.2026.1954055 (PMC13736723; doi:10.3389/fphys.2026.1954055)
Supplement: Supplementary file 1 [file SupplementaryFile1.docx]

**Search strategy**

**PubMed**: ("Pancreatitis"[Mesh] OR pancreatitis) AND chronic AND ("Placebo Effect"[Mesh] OR "Placebos"[Mesh] OR placebo*) AND ("Randomized Controlled Trials as Topic"[Mesh] OR "Randomized Controlled Trial" [Publication Type] OR "randomized controlled trial*" OR RCT)

**Web of Science**: pancreatitis (All Fields) and chronic (All Fields) and placebo* (All Fields) and "randomized controlled trial*" OR RCT

**Embase**: ('pancreatitis'/exp OR pancreatitis:ti,ab,kw)

AND chronic:ti,ab,kw

AND ('placebo effect'/exp OR 'placebo'/exp OR placebo*:ti,ab,kw)

AND ('randomized controlled trial'/exp OR 'randomized controlled trial':de OR 'randomized controlled trial*':ti,ab,kw OR rct:ti,ab,kw)

**Scopus**: TITLE-ABS-KEY(pancreatitis)

AND TITLE-ABS-KEY(chronic)

AND (TITLE-ABS-KEY("placebo effect") OR TITLE-ABS-KEY(placebo*))

AND (TITLE-ABS-KEY("randomized controlled trial") OR TITLE-ABS-KEY("randomized controlled trial*") OR TITLE-ABS-KEY(rct))
